# Physiological and Clinical Aspects of Bioactive Peptides from Marine Animals

**DOI:** 10.3390/antiox11051021

**Published:** 2022-05-22

**Authors:** Sukwasa Chakniramol, Andreas Wierschem, Man-Gi Cho, Khawaja Muhammad Imran Bashir

**Affiliations:** 1 Division of Energy and Bioengineering, Dongseo University, Busan 47011, Korea; punch.chak@gmail.com (S.C.); mgcho@gdsu.dongseo.ac.kr (M.-G.C.); 2 Institute of Fluid Mechanics, Friedrich-Alexander-University Erlangen-Nürnberg, 91058 Erlangen, Germany; andreas.wierschem@fau.de; 3 German Engineering Research and Development Center, LSTME-Busan Branch, Busan 46742, Korea

**Keywords:** anticancer, antioxidant, biopharmaceuticals, clinical, fish, functional foods, in vitro, in vivo, protein hydrolysate

## Abstract

Biological molecules in nutraceuticals and functional foods have proven physiological properties to treat human chronic diseases. These molecules contribute to applications in the food and pharmaceutical industries by preventing food spoilage and cellular injury. Technological advancement in the screening and characterization of bioactive peptides has enabled scientists to understand the associated molecules. Consistent collaboration among nutritionists, pharmacists, food scientists, and bioengineers to find new bioactive compounds with higher therapeutic potential against nutrition-related diseases highlights the potential of the bioactive peptides for food and pharmaceutic industries. Among the popular dietary supplements, marine animals have always been considered imperative due to their rich nutritional values and byproduct use in the food and pharmaceutical industries. The bioactive peptides isolated from marine animals are well-known for their higher bioactivities against human diseases. The physiological properties of fish-based hydrolyzed proteins and peptides have been claimed through in vitro, in vivo, and clinical trials. However, systematic study on the physiological and clinical significance of these bioactive peptides is scarce. In this review, we not only discuss the physiological and clinical significance of antioxidant and anticancer peptides derived from marine animals, but we also compare their biological activities through existing in vitro and in vivo studies.

## 1. Introduction

**Figure 1:**
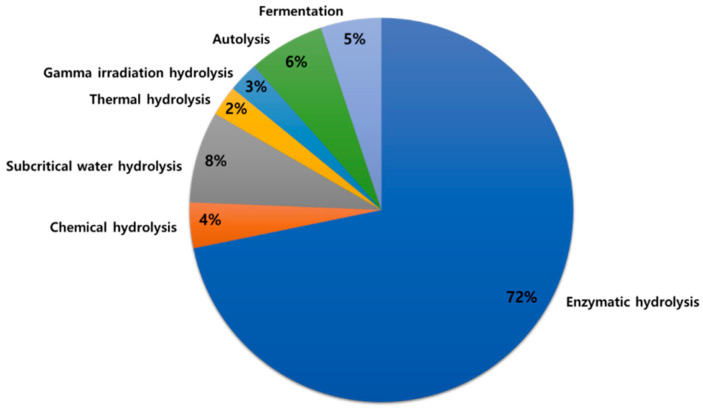
Current methods of producing marine animal-based protein hydrolysates and peptides.

Proteins are important nutrients for sustaining life and supporting normal body functions. Proteins and peptides from marine sources provide a rich source of essential amino acids required for human daily needs [1,2]. Inactive peptides within native proteins require proteolytic release (in vivo digestion) or hydrolysis to reveal biological activity [3,4]. Protein hydrolysis results in the conversion of intact proteins into peptides, usually containing no more than 20 amino acids [4]. Current methods for hydrolysis of marine animal muscle and processing waste byproducts include chemical (acid, alkali, or catalytic) hydrolysis [5], enzymatic hydrolysis [6], gamma irradiation hydrolysis [7], subcritical water hydrolysis [8], thermal hydrolysis [9], autolysis [10], and bacterial fermentation [11] (Figure 1). Among those, enzymatic hydrolysis is the most common method of producing hydrolyzed proteins and peptides. Enzymatic hydrolysis improves solubility, water-binding capacity, and heat stability of myofibrillar proteins, modifies emulsifying and foaming properties of hydrolyzed proteins, as well as improves the nutritional quality of foods [12,13]. Desirable physicochemical properties of a hydrolyzed protein could be achieved by controlling hydrolysis parameters, such as time, pH, temperature, and enzyme concentration [14]. The degree of hydrolysis, on the other hand, affects the size and amino acid profile of peptides, ultimately affecting the taste of the hydrolyzed proteins; for example, bitter taste is associated with a high degree of hydrolysis [15].

Bioactive compounds are either produced naturally from plants, animals, fungi, and microorganisms, such as carotenoids, phenolic compounds, polyphenols, and cordymin [16,17,18,19,20], or generated synthetically, such as propyl gallate, t-butylhydroquinone, butylated hydroxytoluene, and butylated hydroxy anisole [21,22]. Bioactive substances from natural sources have gained much interest in recent years due to consumer’s preferences and health concerns associated with the use of synthetic food additives [23]. The natural protein hydrolysates and peptides isolated from farm animals, such as cows, chickens, and pigs, are relatively cheap and are easily available sources of nutrients [24,25]. However, outbreaks of certain diseases, including avian influenza, mad cow disease, and foot-and-mouth disease, as well as limited use of extracted proteins from pig skin and bones due to religious reasons, have made it obligatory to find alternative sources [26,27]. Recently, the marine-derived protein hydrolysates and bioactive peptides have gained much attention due to their excellent nutritional composition, numerous health benefits and no reported side effects [6,28,29,30,31].

An enormous range of marine-based protein hydrolysates and peptides using different hydrolysis methods have been reported in the literature with significant antioxidant, anticancer, antihypertensive, antimicrobial, and immunomodulatory activities [32,33,34]. Antihypertensive peptides or angiotensin-converting enzyme (ACE) inhibiting peptides have been extensively studied [35]. Due to the side effects, such as taste disturbance and skin rashes, associated with the use of synthetic drugs [36], the interest in finding safer and eco-friendly ACE inhibitory compounds from natural sources has increased [37,38]. The ACE inhibitory properties of the peptides isolated from marine sources, including pacific cod skin [39], sardinella [40], flounder fish [41], and *Takifugu bimaculatus* skin [42], have been reported.

Antimicrobial peptides from marine sources, including algae, sponges, fungi, oysters, and fish, have been reported [19,43,44,45,46,47,48]. Marine antimicrobial peptides are classified into four families based on their structure [49]. These are (i) linear α-helical peptides, (ii) helical or non-helical peptides that are rich in proline, arginine, histidine, and/or glycine, (iii) hairpin-like β sheet or α helical/β-sheet mixed structure peptides with intra-molecular disulfide bonds, and (iv) cyclic peptides [19,49]. In addition, more than 60 antimicrobial peptide drugs are available on the market, and more than 140 peptides are currently in clinical trials [50]. For example, oyster peptide “CgPep33” shows bioactivities against Gram-positive and Gram-negative bacteria and fungi [48].

Immunomodulatory peptides prevent or treat immune-suppressing diseases by improving cytochrome or antibody synthesis and supplementing mucosal immunity in the gastrointestinal tract [51]. The protein hydrolysates and peptides from marine sources have shown significant immunomodulatory properties [52]. For example, a low-molecular-weight peptide isolated from marine *Nibea japonica* skin dramatically improved IgG and IgM levels in blood [53]. Furthermore, due to rising cancer cases, the interest in finding a suitable cancer therapy is consistently gaining attention. Traditional cancer treatments, including surgery, chemotherapy, and radiotherapy, could affect healthy cells [54] and suppress the human immune system [55]. The commercially available drugs from natural sources containing bioactive peptides account for 60% of the available anticancer drugs [56]. It could be due to the ability of the anticancer peptides to penetrate and directly bind to the cancer cells via electrostatic interaction [57]. Recent research has claimed that anticancer compounds from marine sources, especially marine natural bioactive products [58] and nanomaterials, as a safer alternative, allowing targeted treatment to cancer cells and not inducing damage to the healthy cells [59,60].

Peptides isolated from marine sources contain essential amino acids [61] and are used in animal and human nutrition. Due to their rich nutritional profile and bioactive properties, marine peptides have several commercial applications in the food, cosmetics, and pharmaceutical industries [62,63]. The in vitro bioactivities of protein hydrolysates have been discussed in the literature; however, a systematic study on the physiological and clinical significance of marine-based purified bioactive peptides is scarce. Hence, there is a need to critically review the physiological and clinical properties of purified bioactive peptides from marine animals. In this review, we discuss the physiological and clinical significance of antioxidant and anticancer properties of peptides purified from marine animals through selected in vitro, in vivo, and clinical studies.

## 2. Methods

**Figure 2:**
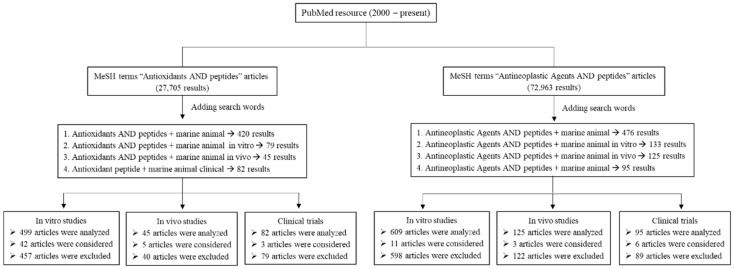
Schematic diagram of search strategy.

The literature search was conducted using the PubMed database from 2000 to present. Articles were mostly written in English (in vitro, in vivo, and clinical studies). The key search using a comprehensive list of MeSH (Medical Subject Headings) terms followed: Antioxidants AND peptides, Antineoplastic Agents AND peptides. Additional search words included “marine animal”, “marine animal in vitro”, “marine animal in vivo”, and “marine animal clinical”. The search strategy and results are presented in a schematic diagram (Figure 2). Articles were selected based on their relevance to the topic of this review. For antioxidant studies, 626 articles were analyzed, 576 articles were excluded, and 50 articles were considered in this review. For anticancer studies, 829 articles were analyzed, 809 articles were excluded, and 20 articles were considered. In addition, references in each article were further reviewed to find any relevant study using ScienceDirect, Wiley Online Library, SpringerLink, and Google Scholar databases. Articles were extracted and sorted using EndNote to prevent duplicate citations.

## 3. Antioxidant Properties

An unbalanced electron in the outermost orbital of reactive oxygen species (ROS) makes these molecules unstable and enables them to react with biological macromolecules, such as proteins, lipids, and DNA [64]. The ROS, including superoxide anion radical (O_2_^•−^), hydrogen peroxide radical (H_2_O_2_), hydroxyl radical (^•^OH), peroxyl radical (ROO^•^), and hydroperoxyl radical (HO_2_^•^), are byproducts produced by mitochondrial aerobic metabolism [65]. An unbalance in the oxidation reaction chain and ROS ratio causes oxidative stress. This leads to the development of chronic diseases, such as diabetes, cardiovascular diseases, Alzheimer’s, neurodegenerative diseases, and particularly cancer [66], which is one of the leading causes of mortality worldwide [67]. The oxidative stress generated by ROS is suppressed by antioxidant defense mechanisms, including endogenous and exogenous defense systems [68]. Endogenous antioxidants, such as catalase (CAT), superoxide dismutase (SOD), glutathione (GSH), and glutathione peroxidase (GPx), and exogenous antioxidants, such as carotenoids, vitamin E, vitamin C, and polyphenols, act synergistically to inhibit oxidation reaction, maintain redox homeostasis and reduce cell deterioration via suppressing cell damage [68,69,70]. Therefore, maintaining a balance in antioxidants/ROS ratio is vital to avoid oxidative stress [71].

The antioxidant effects of peptides isolated from fish and other marine animals have been studied extensively in vitro and in vivo (including animal-based studies), while human clinical trials have rarely been reported. The antioxidant properties of peptides isolated from marine animals are listed in Table 1, Table 2 and Table 3 and are described below.

### 3.1. Antioxidant Effects of Bioactive Peptides from Marine Animals—In Vitro Studies

Proteolytic enzymes generally cleave proteins at a specific cleavage bond resulting in peptides and amino acids of varying sizes [117]. The results of enzyme proteolysis depend on the type of enzyme, temperature, pH, time, and enzyme/substrate ratio [118]. Bashir et al. reported high DPPH^•^ scavenging activity (36.34%) of mackerel (*Scomber japonicus*) peptide, ALSTWTLQLGSTSFSASPM (1049.6 Da), isolated by protamex hydrolysis at 50 °C, pH 8, and in a short time of < 2 h [4]. Zhao and colleagues hydrolyzed Spanish mackerel (*Scomberomorous niphonius*) at 40 °C and pH 5–9 [92]. The resulted peptide, FGYDWW, showed high DPPH^•^, ^•^OH, and O_2_^•−^ scavenging activities of 86.52%, 84%, and 86%, respectively [92]. Similarly, Jang and co-workers observed high DPPH^•^ scavenging activity of 90.66% at 1.0 mg/mL of sandfish peptide, ATSHH (551.25 Da) [104].

The structural features of a peptide, such as size, molecular weight, sequence, and amino acid composition, could directly affect the antioxidant activity of a peptide. The low molecular weight of a peptide (< 1.5 kDa) has been linked with higher ROS radical scavenging activities [4,74,87,95,98]. A low-molecular-weight peptide, TCGGQGR (678 Da) from mackerel byproducts, showed DPPH^•^ scavenging activity of 96% and ABTS^•^ scavenging activity of 100% [95]. The tuna backbone peptide, VKAGFAWTANQQLS (1.5 kDa), showed up to 90% ^•^OH scavenging activity at a concentration of 0.05 mg/mL, and no toxicity to human fetal lung fibroblast cells was reported [73]. Exceptionally, some high molecular weight peptides, such as yellowfin sole peptide, RPNFDLEPPY (13 kDa), have also shown high antioxidant activities [72].

The presence of a hydrophobic amino acid, such as glycine (G), leucine (L), isoleucine (I), and alanine (A) in a peptide sequence could enhance the antioxidant activity of a peptide [4]. These hydrophobic amino acids allow access to the hydrophobic targets, such as cell membrane, and enhance bioavailability [4,119]. Asaduzzaman et al. reported high DPPH^•^ (50%) and ABTS^•^ (40%) scavenging activity of mackerel peptide having glycine [94]. A low-molecular-weight (747 Da) peptide from giant squid (*Dosidicus gigas*), NGLEGLK having glycine, showed reduced cytotoxicity against human embryonic lung fibroblasts at IC_50_ of 304–578 µM [86]. In another study, hoki (*Johnius belengerii*) peptide (1.8 kDa) showed reduced cytotoxicity to human fetal fibroblasts cells at IC_50_ of 17–172 µM and induced ^•^OH scavenging activity [91]. The peptide, CAAP (360 Da) from flounder having alanine, showed potent DPPH^•^ scavenging activity at IC_50_ of 26.89 µM [80]. The sturgeon peptide, GDRGESGPA (845.37 Da), having glycine and alanine, showed DPPH^•^ scavenging activity of 40% [101]. Hairtail surimi peptide, DLYANTVLSGGTTMYPGIADR (2.2 kDa), having leucine, glycine, isoleucine, and alanine, showed DPPH^•^ and ^•^OH scavenging activity of 67.07% and 62.08%, respectively [100]. Furthermore, oyster peptide, LANAK (515.29 Da), showing DPPH^•^ scavenging activity of 83.79%, also contained leucine and alanine [87]. The antioxidant peptides from the skin of horse mackerel, NHREDR (856 Da), and croaker, GNRGFACRHA (1101.5 Da), showed DPPH^•^ scavenging activity of 72.3% and 52.7%, respectively, and ^•^OH scavenging activity of 51.2% and 40.3%, respectively [83]. In another study, peptide—ACFL (518.5 Da), isolated from horse mackerel (*Magalaspis cordyla*) viscera, showed DPPH^•^ and ^•^OH scavenging activity of 57.8% and 45.2%, respectively [93]. These peptides also contained at least one hydrophobic amino acid in their sequences.

The presence of a polar amino acid such as tyrosine (Y) at the C-terminus of a peptide sequence has also been linked with higher antioxidant activities [76,120,121]. The tilapia (*Oreochromis niloticus*) peptides, DCGY (456.12 Da) and NYDEY (702.26 Da), having tyrosine, showed H_2_O_2_ scavenging activity at IC_50_ of 27.6 µg/mL and 38.4 µg/mL, respectively [75]. Similarly, tilapia skin peptide, YGDEY (645.21 Da), showed ^•^OH scavenging activity at IC_50_ of 4.61 µg/mL [76], and tilapia scale peptide, GYDGY (646.23 Da), showing potent DPPH^•^ scavenging activity at IC_50_ of 1.6 µg/mL, contained tyrosine [78]. The Alaska pollock (*Theragra chalcogramma*) peptide, LPHSGY (672 Da), showed high ^•^OH scavenging activity of 35% at a concentration of 53.6 µM and also contained tyrosine [88].

The marine animal-based antioxidant peptides were explored from different marine fish species, where mackerel and tilapia contributed the most. The antioxidant peptides prepared using enzymatic hydrolysis at temperatures of < 50 °C and pH < 9 resulted in higher antioxidant activities. Irrespective of the source of isolation, smaller molecular weight peptides showed higher radical scavenging potential. Interestingly, the presence of a hydrophobic amino acid and/or a polar amino acid, such as tyrosine, in the peptide sequence showed higher scavenging activities. Despite the promising in vitro activities, most of the above-mentioned peptides have not been evaluated under in vivo or clinical conditions.

### 3.2. Antioxidant Effects of Bioactive Peptides from Marine Animals—In Vivo Studies

Nazeer et al. purified the peptide KTFCGRH with a molecular weight of 861 Da from a croaker and studied the antioxidant effects in Wistar rats. A total of 100 µg/kg of purified peptide and 20% ethanol were orally administered to experimental rats for 15 days. The rats fed with purified peptides showed improvement in cellular antioxidant enzyme systems and displayed serum CAT, SOD, and glutathione s-transferase (GST) levels of 283.6, 28.42, and 4.3 U/mg protein, respectively. In contrast, the ethanol-administered rates showed CAT, SOD, and GST levels of 196.4, 15.1, and 1.3 U/mg protein, respectively [110]. The purified peptide from horse mackerel viscera, ACFL with a molecular weight of 518 Da, was evaluated in Wistar rats for 15 days. The results showed that 100 µg/kg of peptide and 20% of ethanol-administered rats showed serum CAT, SOD, and GST levels of 290.8, 29.45, and 3.93 U/mg protein, respectively. In contrast, the ethanol-administered rats showed CAT, SOD, and GST levels of 196.4, 15.1, and 1.3 U/mg protein, respectively [111]. Antioxidant properties of the peptide WHKNCFRCAKCGKSL purified from snakehead murrel (*Channa striatus*) were investigated in wild-type zebrafish. This peptide showed no cytotoxicity to zebrafish larvae at a concentration of 50 µM, and a decrease in malondialdehyde (MDA) levels to 14 µmol/min/mg protein was observed. In contrast, SOD and CAT expressions increased to 22 and 17 U/mg protein, respectively [106].

Hu et al. studied the protective effects of tilapia scales-purified collagen oligopeptides in specific pathogen-free (SPF) Sprague Dawley rats for 30 days [113]. The oral administration of collagen peptides significantly reduced gastric and duodenal ulcer index and MDA content. In comparison, increases in gastric juice pH and expressions of CAT, SOD, and glutathione peroxidase (GPx) were observed [113]. Furthermore, the oligopeptides purified from frigate tuna (*Auxis thazard*) showed hypouricemic properties. Seven days of treatment of SPF hyperuricemic Kunming mice with tuna oligopeptides resulted in increased SOD and CAT levels of 125 and 0.75 U/mg protein, respectively [112].

The purified marine antioxidant peptides were evaluated using different in vivo models, including albino Wistar rats, SPF-SD mice, and wild zebrafish. The study parameters included changes in body weights, body weight gains, levels of intracellular ROS, levels of lipid peroxidation, and expressions of antioxidant enzyme systems, such as SOD, CAT, GST, and GPx. The reported antioxidant peptides showed enhanced expressions of antioxidant enzymes, reduced MDA levels, and helped to recover oxidative stress.

### 3.3. Antioxidant Effects of Bioactive Peptides from Marine Animals—Clinical Trials

Human clinical studies of purified antioxidant peptides from marine animals have rarely been reported. Kim et al. reported skin improving properties of low-molecular-weight collagen peptide (LMWCP) from Sutchi catfish skin (*Pangasius hypophthalmus*) in 40–60-year-old healthy women [114]. Skin hydration was significantly improved (6-fold of placebo), and crow’s feet decreased to 2.81 AU when compared to placebo after 6 weeks of treatment. Average roughness of skin wrinkling—0.17 AU (lower than placebo group) and average roughness of skin elasticity—0.71 AU (higher than placebo group) was noted after 12 weeks. In another study, skin improving properties of CELERGEN^®^ were investigated in 41 adults, including five females. CELERGEN^®^ is a commercial oral supplement that includes marine collagen peptide from the skin of pollock (*Pollachius virens*), Atlantic halibut (*Hippoglossus hippoglossus*), European plaice (*Pleuronectes platessa*), and plant-derived antioxidants [116]. After 4 months of treatment, the thickness and acoustic density of the dermis layer improved to 4133 µm and 6.3, respectively. However, GPx/GST activity and thickness and acoustic density of the epidermis layer were not changed. Another commercial product, GOLD COLLAGEN^®^ ACTIVE, produced from fish collagen, showed skin improving properties when evaluated in 122 subjects [115]. This supplement consists of hydrolyzed fish collagen, vitamins, and other active ingredients. It improved skin elasticity by 40% in the younger age group and reduced joint pain by 43% in the elder age group when compared to the placebo group.

The reported clinical studies on purified antioxidant peptides from marine animals have focused on skin properties, such as skin elasticity, skin hydration, and wrinkling. Two of the above-mentioned products have already been approved and available commercially. Nevertheless, some peptides from marine animals have shown prominent in vitro and in vivo antioxidant activities, and clinical trials are lacking, which need to be performed to better understand their bioactivities.

## 4. Anticancer Properties

Anticancer peptides have been isolated from various marine animals, such as fish, oysters, mussels, and snails. Anticancer peptides are selective to cancer cells and kill cancerous cells by inhibiting angiogenesis, disrupting tubulin-microtubule balance, and apoptosis [122]. The anticancer properties of several peptides from marine animals have been investigated in vitro and in vivo, including in animal-based studies, and some of these peptides have even been used in human clinical trials. The anticancer properties of peptides isolated from marine animals are listed in Table 4, Table 5 and Table 6 and are described below.

### 4.1. Anticancer Effects of Bioactive Peptides from Marine Animals—In Vitro Studies

Anticancer peptides from marine animals, especially the phylum Mollusca, have been extensively reported. The peptide, LKEENRRRRD, isolated from sepia ink (Sepia esculenta), inhibited the proliferation of prostate cancer (PC-3) cells and showed a 3.3-fold increase in early apoptosis as compared to the control [127]. In another study, the peptide, AFNIHNRNLL, from mussel (Mytilus coruscus) inhibited various cancer cell lines, including prostate cancer (PC-3), lung cancer (A-549), and breast cancer (MDA-MB-231) cell lines at LC_50_ of 0.94, 1.41, and 1.22 mg/mL, respectively, and showed no toxicity to the normal cells [125]. The peptide, LANAK (515 Da), isolated from oyster (*Saccostrea cucullata*), not only showed strong antioxidant activity [87], but it also showed significant anticancer activity against human colon cancer (HT-29) cells at IC_50_ of 90 µg/mL and showed no cytotoxicity to normal kidney epithelial cells of the African Green Monkey [87]. Kahalalide F (KF), derived from marine mollusk (*Elysia rufescens*), showed anticancer activity by reducing DNA synthesis in prostate cancer cell lines (DU145, PC3, and LNCaP at IC_50_ of 0.18, 0.07, and 0.26 µM, respectively), breast cancer cell lines (MCF-7, SKBR-3, MDA-MB-231, and BT474 at IC_50_ of 0.28, 0.23, 0.39, and 0.26 µM, respectively), colon cancer cell line (LoVo at IC_50_ of 0.16 µM), and normal cell lines (IMR90, MCF10A, HMEC-1, and HUVEC at IC_50_ of 3.13, 2.44, 1.88 and 1.62 µM, respectively) [131].

Besides mollusks, the anticancer activity of fish has also been claimed. Finless sole peptide, GFFALIPKIISSPLFKTLLSAVGSALSSSGGQE, known as pardaxin, inhibited colony formation in MN-11 cells (<90% inhibition) [126]. The Japanese flounder (*Paralichthys olivaceus*) peptides, RKQCIRKCIRRREPHGKMMIRIRRK and KKYRSQRKIRRMRR-KRKYPSFMQ, induced dose-dependent necrosis in HT-29 cells at a concentration of 500 µM [128]. Spanish mackerel peptides showed a more than 80% survival rate in mouse melanoma cancer (B16F10) cells [129]. The tilapia peptide, GIKCRFCCGCCTPGICGVCCRF-NH_2_, showed a concentration-dependent (50 and 100 µg/mL) inhibition of HeLa and HT1080 cells after 24–72 h treatment. The tilapia peptide inhibited colony formation in HeLa and HT1080 cells by 60% and in HepG2 cells by 50% [130]. Low-molecular-weight antioxidant peptide, FIMGPY (726.9 Da), from skate (Raja porosa) also showed significant anticancer activity against HeLa cells at IC_50_ of 4.81 mg/mL and no toxicity to normal NIH3T3 cells [120].

Recent studies have claimed significant anticancer properties of marine-derived peptides, even at low concentrations, such as anticancer peptides isolated from tuna (*Thunnus tonggol*) [123]. The tuna peptides, LPHVLTPEAGAT and PTAEGGVYMVT, were active against breast cancer (MCF-7) cells at IC_50_ of 8.1–0.7 [123]. The isolated peptides had a molecular weight of 390 Da to 1.4 kDa. In another study, a low-molecular-weight peptide (440 Da) isolated from anchovy sauce inhibited human lymphoma (U937) cells by 50% at 31 µg/mL [124]. Interestingly, these peptides also contained hydrophobic amino acids.

The peptides purified from marine animals, including marine mollusks, tilapia, mackerel, and oysters, showed significant in vitro anticancer activities against breast, leukemia, colon, fibrosarcoma, prostate, and cervical cancers. Interestingly, low-molecular-weight peptides containing hydrophobic amino acids showed high anticancer activities. A similar observation was noted in the case of antioxidant peptides. Irrespective of significant in vitro anticancer activities, in vivo activities of these peptides have hardly been reported.

### 4.2. Anticancer Effects of Bioactive Peptides from Marine Animals—In Vivo Studies

Marine animals from different phyla, especially Mollusca and Porifera, have revealed significant anticancer activities, which have been tested through in vitro and in vivo studies. Venoms of marine cone snails, *genus Conus*, containing more than 100,000 small bioactive peptides, have been used to develop potential therapeutic agents for cardiovascular and nervous systems [133]. The anticancer activity of marine vermivorous cone snail (*Conus vexillum*) venom was investigated by Abdel-Rahman and colleagues using Swiss albino mice injected with Ehrlich’s ascites carcinoma (EAC) cells [133]. Conus venom significantly increased various oxidative stress biomarkers (protein carbonyl content, lipid production, and reactive nitrogen intermediates) of EAC cells after 3–12 h of venom injection. In addition, it significantly reduced CAT and SOD expression levels of EAC cells.

Pardaxin peptide was discovered as a natural agent to inhibit oral squamous cancer [132]. It is derived from finless sole (*Pardachirus marmoratus*) and have been tested on hamster using an oral squamous cell carcinoma model [132]. Pardaxin at a dose of 75 mg/kg reduced tumor volume to more than half. Furthermore, a 500% decrease in serum prostaglandin E2 (a cancer inducer) levels was observed after taking pardaxin with 5-fluorouracil (an antimetabolite used in clinical therapy). In another study, 14 days of treatment with a high dose of synthetic pardaxin (13 µg/mL) showed a 3-fold reduction in tumor volume of MN-11 cells in C57BL/6 mice, while no side effects were observed in the normal cells [126].

### 4.3. Anticancer Effects of Bioactive Peptides from Marine Animals—Clinical Trials

The anticancer activity of marine animal-derived individual peptides has not been claimed through clinical trials; however, two decades ago, some anticancer compounds containing marine animal-derived peptides were reported, which are currently known as natural chemotherapeutic agents such as Kahalalide F (KF) [140]. KF is a depsipeptide with a composition ranging from tripeptide to tridecapeptide [141]. KF was first isolated from marine mollusk (*Elysia rufescens),* and it is currently in clinical phase II. KF has shown significant antitumor activities in prostate cancer patients at a recommended dose of 560 µg/m^2^ [135]. Another clinical study on 38 cancer patients (colorectal cancer, melanoma cancer, breast cancer, and others) recommended a KF dose of 650 µg/m^2^ [134]. However, dose-limiting toxicities were observed after 1 h intravenous infusion [134]. Martín-Algarra and co-workers confirmed 650 µg/m^2^ as the safe recommended dose of KF and suggested KF peptide as a safe chemotherapeutic agent [136]. The KF-treated cancer patients showed an average overall survival of 10.8 months, non-cumulative toxicity, and no side effects such as leukopenia or thrombocytopenia when used with other compounds. However, the use of KF alone for cancer treatment has not yet been documented for advanced malignant patients.

Another marine mollusk (*Dolabella auricularia*) peptide, DOLA-10, currently in clinical trial phase II [138], showed no changes in tumor volume in clinical phase I trials on 22 cancer patients at a maximum dose of 300 µg/m^2^ [137]. Pitot et al. recommended a dose of 400 µg/m^2^ for patients requiring less than two chemotherapies and 325 µg/m^2^ for patients requiring more than two chemotherapies [142]. Therefore, DOLA-10 at a dose of 400 µg/m^2^ was used in clinical phase II [138]. Krug et al. studied 400 µg/m^2^ of DOLA-10 in 10 NSCLC patients [138], where only 2 patients had a stable disease, 3 patients had grade-4 neutropenia, and 2 patients had grade-3 hyperglycemia. Later, Margolin and colleagues again studied DOLA-10 at 400 µg/m^2^ [139] and observed the pharmacokinetic profile at the total body clearance and volume of distribution in the body at a steady state of 2.61 ± 1.9 L/h/m^2^ and 28.4 ± 13 L/m^2^. The above-mentioned studies highlight the potential anticancer properties of marine animal-derived peptides; however, further clinical studies are needed to better understand their mechanism of action.

## 5. Conclusions and Future Perspectives

The isolation, purification, and characterization of bioactive peptides from marine sources have significantly increased in the last few years. The marine processing byproducts and the underutilized marine organisms, especially fish, have been highlighted as potential sources of bioactive peptides. Different methods, such as proteolytic hydrolysis, chemical hydrolysis, subcritical water hydrolysis, and autolysis, have been used to isolate peptides from marine animals, with enzymatic hydrolysis being the most used. However, the emerging techniques have not been well used. Hence, there is a need to explore the emerging innovative technologies, such as pulsed electric field, ohmic heating, and high hydrostatic pressure processing, to produce peptides with significant bioactivities against human diseases. Even though several studies have reported the bioactivities of marine-based purified peptides, more research is needed to explore their full potential for food, pharmaceutical, and cosmetic industries. While interesting studies on the use of marine animal-based antioxidant and anticancer peptides have been reported in vitro, their bioavailability, functionality, shelf life, and long-term stability need to be addressed through in vivo studies for further use and large-scale production. Furthermore, to use these peptides for human consumption as nutraceuticals, bio-functional foods, or natural additives in food and healthcare products and in biopharmaceuticals, their mechanism of action should be determined through clinical studies to better understand their physiological properties.

## Figures and Tables

**Table 1:** Antioxidant effects of bioactive peptides from marine animals—in vitro studies.

Peptide Name/Sequence	Size of Peptide (Da)	Source	Cell Line	Analysis	Major Findings	Reference
RPNFDLEPPY	13 K	Yellowfin sole (*Limanda aspera*)	-	TBARS assay, HPLC	- TBARS activity of 0.04 mg MDA/kg	[72]
VKAGFAWTANQQLS	1519	Tuna—backbone	MRC-5 and ECV304	DPPH^•^, O_2_^•−^, and ^•^OH scavenging assay, lipid peroxidation inhibition assay, FPLC, MTT assay, Q-TOF MS	- DPPH^•^ scavenging activity of 65% at 0.05 mg/mL - ^•^OH scavenging activity of 90% at 0.05 mg/mL - O_2_^•−^ scavenging activity of 67% at 1 mg/mL	[73]
(i) GAERP (ii) GEREANVM (iii) AEVG	(i) 528.57 (ii) 905 (iii) 374.4	Spotless smooth hound—cartilage(*Mustelus griseus*)	HepG2	DPPH^•^, ^•^OH, O_2_^•−^, and ABTS^•^ scavenging activity, lipid peroxidation inhibition assay, MTT assay	- Peptide (i) showed O_2_^•−^ scavenging activity of 80% - Peptide (ii) showed DPPH^•^ and ABTS^•^ scavenging activity of 70% and 90%, respectively - Peptide (iii) showed ^•^OH scavenging activity of 95%	[74]
(i) DCGY (ii) NYDEY	(i) 456.12 (ii) 702.26	Tilapia—frame (*Oreochromis niloticus*)	-	DPPH^•^, ^•^OH, O_2_^•−^, and H_2_O_2_ scavenging activity	- Peptide (i) showed H_2_O_2_ scavenging activity at IC_50_ of 27.6 µg/mL	[75]
(i) EGL (ii) YGDEY	(i) 317.33 (i) 645.21	Tilapia—skin (*Oreochromis niloticus*)	-	DPPH^•^, ^•^OH, and O_2_^•−^ scavenging activity, Q-TOF MS, RP-HPLC	- Peptide (i) showed ^•^OH scavenging activity at IC_50_ of 4.61 µg/mL - Peptide (ii) showed ^•^OH scavenging activity at IC_50_ of 6.45 µg/mL	[76]
(i) GH (ii) ED (iii) DHG (iv) EPPF (v) KPFGSGAT	-	Tilapia—skin (*Oreochromis niloticus*)	Erythrocytes	DPPH^•^, ^•^OH, and O_2_^•−^ scavenging activity, HPLC, ESI-MS/MS, hemolysis assay	- Peptide (v) showed ^•^OH scavenging activity of 22.5%	[77]
(i) GYDGY (ii) EPGKSGEQGAPGEAGAP	(i) 646.23 (ii) 1538.7	Tilapia—scales (*Oreochromis niloticus*)	-	DPPH^•^ and ABTS^•^ scavenging activity, FRAP assay, HPLC, LC/ESI-MS/MS	- DPPH^•^ scavenging activity at IC_50_ of 1.6 µg/mL	[78]
FLNEFLHV	1018.48	Salmon—pectoral fin	-	DPPH^•^ and ABTS^•^ scavenging activity, FRAP assay	- DPPH^•^ scavenging activity at IC_50_ of 486 µM - ABTS^•^ scavenging activity at IC_50_ of 152 µM - FRAP activity at IC_50_ of 0.225 µM	[79]
(i) VCSV (ii) CAAP	(i) 406 (ii) 360	Flounder fish (*Paralichthys olivaceus*)	Vero	DPPH^•^ scavenging activity	- Peptide (ii) showed potent DPPH^•^ scavenging activity at IC_50_ of 26.89 µM	[80]
(i) AAVPSGASTGIYEALELR (ii) NPLLEAFGNAK	(i) 1805.03 (ii) 1173.34	Purple sea urchin—gonad (*Strongylocentrotus nudus*)	-	DPPH^•^ scavenging activity	- Both peptides showed DPPH^•^ scavenging activity of 95%	[81]
(i) FIMGPY (ii) GPAGDY (iii) IVAGPQ	(i) 726.9 (ii) 578.58 (iii) 583.69	Skate—cartilage (*Raja porosa*)	-	DPPH^•^, ^•^OH, O_2_^•−^, and ABTS^•^ scavenging activity, lipid peroxidation inhibition assay	- Peptide (i) showed DPPH^•^, ^•^OH, and O_2_^•−^ scavenging activity of 85%, 85%, and 90%, respectively - Peptide (ii) showed ABTS^•^ scavenging activity of 88%	[82]
GNRGFACRHA	1101.5	Croaker—skin (*Otolithes ruber*)	-	DPPH^•^ and ^•^OH scavenging activity, lipid peroxidation inhibition assay, Fe^2+^ chelating activity, IEC, GPC, ESI-MS/MS	- DPPH^•^ scavenging activity of 52.7% - ^•^OH scavenging activity 40.3%	[83]
(i) DGPEGR (ii) GPEGPMGLE (iii) EGPFGPEG (iv) YGPDGPTG (v) GFIGPTE (vi) IGPLGA	(i) 629.61 (ii) 885.95 (iii) 788.96 (iv) 762.75 (v) 733.8 (vi) 526.61	Redlip Croaker—scales *(Pseudosciaena polyactis)*	HepG2	DPPH^•^, ^•^OH, and O_2_^•−^ scavenging activity, ROS levels, lipid peroxidation inhibition assay, IEC, gel filtration chromatography, RP-HPLC	- Peptide (ii) showed O_2_^•−^ scavenging activity of 86% - Peptide (iii) showed DPPH^•^ and ^•^OH scavenging activity of 90% and 87%, respectively	[84]
(i) GPE (ii) GARGPQ (iii) GFTGPPGFNG	(i) 301.3 (ii) 584.64 (iii) 950.03	Scalloped Hammer—cartilage (*Sphyrna lewini*)	-	DPPH^•^, ABTS^•^, ^•^OH, and O_2_^•−^ scavenging activity, lipid peroxidation inhibition assay, IEC, GPC, RP-HPLC, Q-TOF MS	- Peptide (i) showed O_2_^•−^ scavenging activity of 90% - Peptide (ii) showed ^•^OH scavenging activity of 88% - Peptide (iii) showed DPPH^•^ and ABTS^•^ scavenging activity of 75% and 80%, respectively	[85]
(i) NADFGLNGLEGLA (ii) NGLEGLK	(i) 1307 (ii) 747	Giant squid (*Dosidicus gigas*)	MRC-5	^•^OH, and O_2_^•−^ scavenging activity, carbon-centered radical scavenging assay, Q-TOF MS, lipid peroxidation inhibition assay, MTT assay	- Peptide (i) showed lipid peroxidation inhibition of 18.27% - Peptide (ii) showed lipid peroxidation inhibition of 23.32% - Peptide (ii) showed carbon-centered radical, ^•^OH, and O_2_^•−^ scavenging activity at IC_50_ of 304.67, 428.54, and 573.83 µM, respectively	[86]
(i) LANAK (ii) PSLVGRPPVGKLTL (iii) VLVLLEHPVL	(i) 515.29 (ii) 1432.89 (iii) 1145.75	Oyster (*Saccostrea cucullata*)	Vero	DPPH^•^ scavenging activity, Q-TOF MS, UPLC, GPC	- Peptide (i) showed DPPH^•^ scavenging activity of 83.79% - Peptide (ii) showed DPPH^•^ scavenging activity of 75.48% - Peptide (iii) showed DPPH^•^ scavenging activity of 76.62%	[87]
LPHSGY	672	Alaska pollock —frame (*Theragra chalcogramma*)	-	^•^OH scavenging activity, HPLC	- ^•^OH scavenging activity of 35% at 53.6 µM	[88]
(i) GEHGPHGPHGPHGPHG (ii) GPHGPHGPHGPHG	-	Alaska pollock —skin	Ac2F	TBAR assay, MTT assay	- Peptide (ii) showed higher antioxidant activity than peptide (i)	[89]
HGPLGPL	797	Hoki—skin (*Johnius belengerii*)	Hep3B	DPPH^•^ and O_2_^•−^ scavenging activity, SOD, GPx, and CAT activity	- O_2_^•−^ scavenging activity of 97.65 ± 0.43% - Carbon-centered radical scavenging activity of 95.12 ± 0.85% - DPPH^•^ scavenging activity of 80.09 ± 0.31%- SOD activity of 92.8%- GPx activity of 60.78% - CAT activity of 35%	[90]
GSTVPERTHPACPNFN	1801	Hoki—frame (*Johnius belengerii*)	MRC-5	DPPH^•^, ^•^OH, ROO^•^, and O_2_^•−^ scavenging activity, IEC, HPLC, Q-TOF MS, MTT assay	- DPPH^•^, ^•^OH, ROO^•^, and O_2_^•−^ scavenging activity at IC_50_ of 41.37, 17.77, 18.99, and 172.10 µM, respectively	[91]
(i) VKQNAVGIDLGTTY (ii) IPTNDLAPGQFR (iii) IANLAATDIIF (iv) LIGLPHDQPQIL (v) NANDALVDLPTPASITAM (vi) AHVEAPIAGSM (vii) ALSTWTLQLGSTSFSASPM (viii) LGTLLFIAIPI (ix) GDLLNEQSLM (x) LVGEVPGARY	(i) 756.39 (ii) 681.34 (iii) 597.82 (iv) 688.88 (v) 931.95 (vi) 557.77 (vii) 1049.46 (viii) 641.85 (ix) 576.27 (x) 547.29	Chub mackerel (*Scomber japonicus*)	-	DPPH^•^ scavenging activity, FRAP assay, SOD-like activity, MALDI-TOF MS/MS, RP-HPLC	- Peptide (vii) showed DPPH^•^ scavenging activity of 36.34 ± 4.64% - Peptide (viii) showed SOD-like activity of 28.94 ± 4.19%	[4]
(i) PELDW (ii) WPDHW (iii) FGYDWW (iv) YLHFW	(i) 658.72 (ii) 739.81 (iii) 872.93 (iv) 764.90	Spanish mackerel (*Scomberomorus niphonius*)	-	DPPH^•^, ^•^OH, and O_2_^•−^ scavenging activity, lipid peroxidation inhibition assay, plasmid DNA protective assay	- Peptide (iii) showed DPPH^•^, ^•^OH, and O_2_^•−^ scavenging activity of 86.52%, 84%, and 86%, respectively	[92]
NHREDR	856	Horse mackerel—skin (*Magalaspis cordyla*)	-	DPPH^•^ and ^•^OH scavenging activity, lipid peroxidation inhibition assay, Fe^2+^ chelating activity, IEC, GPC, ESI-MS/MS	- DPPH^•^ scavenging activity of 72.3% - ^•^OH scavenging activity 51.2%	[83]
ACFL	518.5	Horse mackerel—viscera (*Magalaspis cordyla)*	-	Lipid peroxidation inhibition assay, DPPH^•^, and ^•^OH scavenging activity, FPLC, Q-TOF MS	- DPPH^•^ scavenging activity of 57.8 ± 1.4% - ^•^OH scavenging activity of 45.2 ± 2.0%	[93]
(i) RSDGSRIRF (ii) MIQMQTKLK (iii) KDAAADKAEDVKD (iv) KEGLGKLTGNEKL (v) RRYANIGDVIKY (vi) KQLDTLGNDKGRL (vii) KDAVEDKVEDAKE (viii) KKGDVYDAVVVRT (ix) RSAQFMKIVSLAPEVL (x) RHAEVVASIKA (xi) RSVDPGSPAARS (xii) MKTAQELRV (xiii) KNLLTGSASESVYKA	(i) 789.36 (ii) 1120.26 (iii) 1132.10 (iv) 1145.09 (v) 1147.08 (vi) 1216.11 (vii) 1218.11 (viii) 1220.10 (ix) 1632.92 (x) 952.30 (xi) 954.29 (xii) 957.27 (xiii) 1368.13	Mackerel—skin (*Scomber japonicus*)	-	MALDI-TOF MS/MS, DPPH^•^, and ABTS^•^ scavenging activity, FRAP assay, Fe^2+^ chelating assay, FTIR assay	- Peptides (i–ix) showed DPPH^•^ and ABTS^•^ scavenging activity of 50%–98% - Peptides (x–xiii) showed DPPH^•^ and ABTS^•^ scavenging activity of 65%–90% - Peptides (i–ix) and (x–xiii) showed Fe_2_^+^ chelating activity of 55% and 65%, respectively	[94]
(i) KTKATLARM (ii) KRMDLARI (iii) KVTFNRKQ (iv) KATLARMARG (v) RMARGAMVRF (vi) RFVFIYQH (vii) MKIIIAPAKK (viii) MADAELEAIRQ (ix) KTLWHCSDKL	(i) 759.12 (ii) 761.11 (iii) 763.14 (iv) 888.62 (v) 890.61 (vi) 952.29 (vii) 984.21 (viii) 986.21 (ix) 989.21	Mackerel—bone (*Scomber japonicus*)	-	MALDI-TOF MS/MS, DPPH^•^, and ABTS^•^ scavenging activity, FRAP assay, Fe^2+^ chelating assay, FTIR assay	- DPPH^•^ and ABTS^•^ scavenging activity of 58% and 42%, respectively - Fe^2+^ chelating activity of 52%	[94]
(i) TCGGQGR (ii) KEAGAFIDR	(i) 678 (ii) 1006	Mackerel—byproducts (*Scomber japonicus*)	-	Protease activity, MALDI-TOF MS/MS, DPPH^•^, and ABTS^•^ scavenging activity	- ABTS^•^ scavenging activity of 100% - DPPH^•^ scavenging activity of 96.04 ± 0.19%	[95]
(i) FWKVV (ii) FMPLH	(i) 611.66 (ii) 1092.23	Chinese drum *(Miichthys miiuy)*	HUVECs	Hoechst 33,342 staining assay, SOD and GPx levels, DNA oxidative damage by H_2_O_2_	- Peptide (i) showed H_2_O_2_-induced DNA inhibition damage of 75.89% - Peptide (ii) showed H_2_O_2_-induced DNA inhibition damage of 70.03% - Peptide (i) showed SOD level of 180.62 U/mg protein - Peptide (i) showed GSH-Px level of 38.67 U/mg protein	[96]
Purified peptide fraction—II	-	*Exocoetus volitans*	Vero	DPPH^•^, ^•^OH, and O_2_^•−^ scavenging activity, lipid peroxidation inhibition assay, MTT assay, IEC, HPLC	- DPPH^•^ scavenging activity of 51.1% - ^•^OH scavenging activity of 56.6% - O_2_^•−^ scavenging activity of 50.3%	[97]
LVPVAVF	743.45	Silver carp (*Hypophthalmichthys molitrix*)	Caco-2	DPPH^•^ scavenging activity, lipid peroxidation inhibition assay, intracellular ROS, SE-HPLC, RP-HPLC, ORAC, FRAP	- DPPH^•^ scavenging activity at EC_50_ of 0.65 mg/mL - ROS inhibitory capacity of 27.23%	[98]
(i) GSGGL (ii) GPGGFI (iii) FIGF	(i) 389.41 (ii) 46.63 (iii) 435.52	Bluefin leatherjacket—skin (*Navodon septentrionalis*)	-	DPPH^•^, ^•^OH, and O_2_^•−^ scavenging activity, metal chelating activity, RP-HPLC	- Peptide (iii) showed DPPH^•^, ^•^OH, and O_2_^•−^ scavenging activity at EC_50_ of 0.118, 0.073, and 0.311 mg/mL, respectively	[99]
DLYANTVLSGGTTMYPGIADR	2214.06	Hairtail surimi	-	DPPH^•^ and ^•^OH scavenging activity, TAO assay, gel filtration chromatography, RP-HPLC, LC-MS/MS	- DPPH^•^ scavenging activity of 67.07% - ^•^OH scavenging activity of 62.08% - TAO activity of 4.41 U/mL	[100]
GDRGESGPA	845.37	Sturgeon—skin (*Acipenser schrencki*)	-	Fe^2+^ chelating activity, DPPH^•^ scavenging activity, SP-RP-HPLC, LC-MS/MS	- DPPH^•^ scavenging activity of 40%	[101]
KGFR	506	Round scad (*Decapterus maruadsi*)	-	DPPH^•^ and ^•^OH scavenging activity, FRAP, gel filtration chromatography, RP-HPLC, LC-MS/MS	- DPPH^•^ scavenging activity at IC_50_ of 0.13 mg/mL	[102]
(i) EDIVCW (ii) MGPVW (iii) YWDAY	(i) 763.82 (ii) 660.75 (iii) 793.75	Monkfish (*Lophius litulon*)	HepG2	DPPH^•^, ^•^OH, H_2_O_2,_ and O_2_^•−^ scavenging activity, ROS levels, lipid peroxidation inhibition assay, Q-TOF MS, MTT assay	- Peptide (i) showed DPPH^•^ scavenging activity of 83% - Peptide (iii) showed ^•^OH and O_2_^•−^ scavenging activity of 85% and 80%, respectively - Peptide (iii) showed SOD, GPx, and CAT levels of 175, 45, and 22.5 U/mg protein, respectively	[103]
ATSHH	551.25	Sandfish (*Arctoscopus japonicus*)	-	DPPH^•^, ^•^OH, and O_2_^•−^ scavenging activity, RP-HPLC, Q-TOF MS	- DPPH^•^ scavenging activity of 90.66% at 1.0 mg/mL	[104]
(i) SLGGASGSTAMQAK (ii) LSGGASGSTAMQAK (iii) NASGSTAMLQAVDNAYAR (iv) QASGSTAMKQAVDNATAR (v) FPGDHDR	-	Greater weever (*Trachinus draco*)	-	RP-HPLC, CUPRAC, Cu^2+^ chelating activity, RP-HPLC, nano-ESI-MS/MS	- Peptide (v) showed TAC activity of 68.2 ± 0.05 μg/mL—ascorbic acid equivalent/μg peptide - Peptide (v) showed Cu^2+^ chelating activity of 153 ± 0.31%/µg peptide	[105]
Purified peptide fraction—I	-	*Nemipterus japonicus*	Vero	DPPH^•^, ^•^OH, and O_2_^•−^ scavenging activity, lipid peroxidation inhibition assay, MTT assay, IEC, HPLC	- DPPH^•^ scavenging activity of 48.7% - ^•^OH scavenging activity of 51.2% - O_2_^•−^ scavenging activity of 44.2%	[97]
WL15 (WHKNCFRCAKCGKSL)	-	Snakehead murrel *(Channa striatus*)	HDF	DPPH^•^, ABTS^•^, O_2_^•−^, and H_2_O_2_ scavenging activity, cytotoxicity assay	- DPPH^•^, ABTS^•^, O_2_^•−^, and H_2_O_2_ scavenging activity of 53%, 50.6%, 60%, and 64.6%, respectively - No toxicity to HDF cells up to a concentration of 50 mM - Decreased intracellular ROS levels by induced H_2_O_2_ exposure	[106]
WL15	-	Snakehead murrel *(Channa striatus*)	L6 myoblast	Intracellular ROS level, lipid peroxidation inhibition assay, SOD and CAT assay, MTT assay	- SOD and CAT levels of 20 and 17 U/mg protein, respectively, at 50 mM	[107]
GGFDMG	582	Japanese flounder—skin (*Paralichthys olivaceus*)	RAW264.t	FPLC, HPLC, DPPH^•^, cell viability assay, lipid peroxidation inhibition assay, radical-mediated damage to membrane lipids, DNA and protein	- Protected the radical-mediated damage of membrane lipids, DNA, and protein - Regulated SOD-1, GSH, and CAT expressions	[108]
FTGML	-	Grass carp—scales (*Latrunculia magnifica*)	B16F10	CCK-8 assay, apoptosis rate	- Potent activity against mouse melanoma cancer cells - Cell viability of > 80% - Cell apoptosis increased from 3% to 25%	[109]

AAPH: 2,2′-Azobis (2-amidinopropane) dihydrochloride; ABTS^•^: 2,2′-Azino-bis (3-ethylbenzthiazoline-6-sulfonic acid) radical; Ac2F: Donryu rat liver cells; CAT: Catalase; CUPRAC: Cupric reducing antioxidant capacity; DPPH^•^: 2,2-Diphenyl-1-picrylhydrazyl radical; ECV304: Human endothelial cells; ESI-MS/MS: Electrospray ionization-mass spectrometry; FPLC: Fast protein liquid chromatography; FRAP: Ferric reducing antioxidant power; FTIR: Fourier transform infrared spectroscopy; GPC: Gel permeation chromatography; GPx: Glutathione peroxidase; GSH: Glutathione; IEC: Ion-exchange chromatography; HepG2: Human hepatocellular liver carcinoma cells; Hep3B: Human hepatoma cells; HO^•^: Hydroxyl peroxide radical; HPLC: High-performance liquid chromatography; H_2_O_2_: Hydrogen peroxide; IC50: The half maximal inhibitory concentration; LC-MS/MS: Liquid chromatography-tandem mass spectrometry; LC/ESI-MS/MS: Liquid chromatography-electrospray ionization-mass spectrometry; L6 myoblast: Immortalized rat skeletal (L6) myoblast cells; MALDI-TOF MS/MS: Matrix-assisted laser desorption ionization-time of flight mass spectrometry; MDA: Malonaldehyde; MRC-5: Human embryonic lung fibroblast cells; MTT: 3-(4,5-dimethylthiazol-2-yl)-2,5-diphenyl tetrazolium bromide; nano-ESI-MS/MS: Nano liquid chromatography-tandem mass spectrometry; ORAC: Oxygen radical absorbance capacity; O_2_^•−^: Superoxide radical; Q-TOF MS: Quadrupole time of flight mass spectrometry; RAW264.t: Mouse monocyte cells; ROO^•^: Peroxyl redical; RP-HPLC: Reversed-phase high-performance liquid chromatography; SEC: Size-exclusion chromatography; SP-RP-HPLC: Semipreparative reversed-phase high-performance liquid chromatography; SOD: Superoxide dismutase; TAO: Total antioxidant capacity; TBARS: Thiobarbituric acid reactive substances; UPLC: Ultra-performance liquid chromatography; Vero: Kidney epithelial cells of the African Green Monkey.

**Table 2:** Antioxidant effects of bioactive peptides from marine animals—in vivo studies.

Peptide Name/Peptide Containing Compound	Size of Peptide (Da)	Source	Organism	Analysis	Major Findings	Reference
WL15	-	Snakehead murrel *(Channa striatus*)	Adult zebrafish (wild-type, AB strain; 4 months old)	ROS production assay, cytotoxicity assay, caspase 3, SOD, CAT, GST, GPx, and GCS expressions	- No development toxicity to zebrafish larvae even at higher tested concentration (50 μM) - Attenuated caspase 3 activation and reduced MDA levels - Enhanced SOD, CAT, GST, GPx, and GCS expressions	[106]
KTFCGRH	861.6	Croaker (*Otolithes ruber*)	9 Adult albino Wistar male rats (body weight range: 150–180 g)	CAT, GST, and SOD activity	- CAT activity of 283.6 U/mg protein - GST activity of 4.3 U/mg protein - SOD activity of 28.42 U/mg protein	[110]
ACFL	518.5	Horse mackerel—viscera (*Magalaspis cordyla*)	9 adult albino Wistar male rats (body weight range: 150–180 g)	CAT, SOD, and GST activity	- CAT activity of 290.8 U/mg protein - GST activity of 3.93 U/mg protein - SOD activity of 29.45 U/mg protein	[111]
Oligopeptide	1000	Frigate tuna *(Auxis thazard)*	60 Kunming SPF-SD male mice 4 weeks old (average body weight: 20 ± 2 g)	Body weight and organ index, XOD, MDA, ADA, SOD, and CAT activity, URAT1, GLUT9, OAT1, and ABCG2 expressions	- SOD activity of 125 U/mg protein - CAT activity of 0.75 U/mg protein - MDA levels of 0.75 nmol/mg - XOD activity of 13 U/g protein - ADA activity of 11 U/g protein	[112]
Collagen oligopeptides	<1000	Tilapia—scales	72 SPF-SD male rats (6–8 weeks old)	SOD, GPx, CAT, and MDA activity	- Gastric tissue showed SOD activity of 91.52 ± 16.5 U/mg protein, CAT activity of 22.24 ± 5.10 U/mg protein, GPx of 37.22 ± 8.65 U/mg protein, and MDA levels of 0.46 ± 0.16 nmol/mg - Duodenal tissue showed SOD activity of 21.38 U/mg protein, CAT activity of 85.45 U/mg protein, GPx activity of 51.9 U/mg protein, and MDA levels of 0.75 nmol/mg	[113]

ABCG2: ATP-binding cassette subfamily G2; ADA: Adenosine deaminase activity; CAT: Catalase; GCS: γ-glutamyl cysteine synthetase; GLUT9: Glucose transporter; GPx: Glutathione peroxidase; GST: Glutathione S-transferase; MDA: Malondialdehyde; OAT1: Organic anion transporter; ROS: Reactive oxygen species; SPF-SD rats: Specific pathogen-free Sprague Dawley rats; SOD: Superoxide dismutase; URAT1: Urate transporter; XOD: Xanthine oxidase.

**Table 3:** Antioxidant effects of bioactive peptides from marine animals—clinical trials.

Peptide/Peptide Containing Compound	Source	Subject	Analysis	Major Findings	Phase	Reference
Low-molecular-weight collagen peptide (LMWCP)	Sutchi catfish—skin (*Pangasius hypophthalmus*)	64 healthy male adults (40–60 years old)	Skin hydration, wrinkling, and elasticity	- Moisture content improved from 50.12 (placebo group) to 60.00 after 6-week treatment - Crow’s feet visual grade was higher than placebo group (0.04 AU) - Average roughness of wrinkling was lower than placebo group (0.02 AU) - Average roughness of elasticity was higher than placebo (0.07 AU)	I	[114]
GOLD COLLAGEN^®^ ACTIVE Hydrolyzed fish collagen complex (Approved and patent granted in Italy: 0001413152)	Fish collagen	122 adults (91 male, 29 female) (21–70 years old)	Skin elasticity, histological examination of skin, SPQs, joint health questionnaire	- Younger age group showed 40% increase in skin elasticity as compared to placebo group after 90 days application - Elder age group showed 43% reduction in joint pain score as compared to placebo group	Approved	[115]
CELERGEN^®^ Marine collagen peptides combined with plant peptides	Skin of *Pollachius virens*, *Hippoglossus hippoglossus*, and *Pleuronectes platessa,*	41 adults (36 male, 5 female) (37–72 years old)	Plasma levels of nitrites/nitrates, MDA, Cu- and Zn-SOD3, facial skin properties	- Thickness of dermis layer increased from 3900 ± 31 µm (before treatment) to 4133 ± 28 µm (after treatment) - Acoustic density of dermis layer increased from 5.1 ± 0.2 (before treatment) to 6.3 ± 0.1 (after treatment)	Approved	[116]

AU: Arbitrary units; MDA: Malondialdehyde; Cu-SOD: Copper superoxide dismutase; SPQs: Self-perception questionnaires; Zn SOD: Zinc superoxide dismutase.

**Table 4:** Anticancer effects of bioactive peptides from marine animals—in vitro studies.

Peptide Name/Sequence	Size of Peptide (Da)	Source	Cell Line	Analysis	Major Findings	Reference
(i) LPHVLTPEAGAT (ii) PTAEGGVYMVT	(i) 1206 (ii) 1124	Tuna fish *(Thunnus tonggol*)	MCF-7	Antiproliferative activity, MTT assay	- Potent activity against human breast cancer cells - Peptide (i) showed IC_50_ of 8.1 µM - Peptide (ii) showed IC_50_ of 0.7 µM	[123]
Peptide fraction-AobsII	440.9	Anchovy—sauce	U937	MTT assay, DNA fragmentation analysis, cell cycle analysis, silica gel chromatography	- Potent activity against human leukemia cancer cells - The IC_50_ of 31 µg/mL	[124]
LANAK	515.29	Oyster (*Saccostrea cucullata*)	HT-29	MTT assay, AO/EtBr staining, comet assay	- Potent activity against human colon cancer cells - The IC_50_ of 60.21 µg/mL after 72 h	[87]
AFNIHNRNLL	-	Mussel *(Mytilus coruscus)*	PC-3, A549, MDA-MB-231	Apoptosis assay, IEC, HPLC	- Potent activity against human prostate, lung, and breast cancer cells - Apoptosis of PC-3 at LC_50_ of 0.94 mg/mL - Apoptosis of A549 at LC_50_ of 1.41 mg/mL - Apoptosis of MDA-MB-231 LC_50_ of 1.22 mg/mL	[125]
Pardaxin	-	Finless sole *(Pardachirus marmoratus*)	MN-11	MTS assay, membrane structure examination, apoptosis assay	- Potent activity against mouse fibrosarcoma cancer cells - More than 90% inhibition in MN-11 colony formation	[126]
LKEENRRRRD	1371.53	Sepia—ink (*Sepia esculenta*)	PC-3	Apoptosis assay, AO/EtBr staining, Western blot	- Potent activity against human prostate cancer cells - An early apoptotic increased from 8.85% to 29%	[127]
(i) RKQCIRKCIRRREPHGKMMIRIRRK (ii) KKYRSQRKIRRMRRKRKYPSFMQ	-	Japanese flounder (*Paralichthys olivaceus*)	HT-29	MTT assay, qRT-PCR	- Potent activity against human colon cancer cells - Appearance of a large number of necrotic cells at a concentration of 500 µM	[128]
(i) T-L-R (ii) T-I-R (iii) L-D-D-L (vi) I-D-D-L (v) L-D-D-I (vi) I-D-D-I (vii) L-Q-L-E (viii) L-Q-I-E (ix) I-Q-L-E (x) I-Q-I-E (xi) L-N-L-T (xii) I-N-L-T (xiii) L-N-I-T (xiv) I-N-I-T (xv) A-L-D-L-H (xvi) A-I-D-L-H (xvii) A-L-D-I-H (xviii) A-I-D-I-H	(i) 388.46 (xi) 459.54 (xv) 567.64	Spanish mackerel *(Scomberomorus niphonius)*	B16F10	MTT assay, UPLC, UPLC-ESI-MS/MS	- Potent activity against mouse melanoma cancer cells - Inhibited the survival rate of B16-F10 cells up to 80.2%	[129]
FIMGPY	726.9	Skate—cartilage (*Raja porosa*)	HeLa	MTT assay, Western blot	- Potent activity against human cervical cancer cells - The IC_50_ of 4.81 mg/mL	[120]
GIKCRFCCGCCTPGICGVCCRF-NH2 (Amidated C-terminus)	-	Tilapia (*Oreochromis mossambicus*)	HeLa, HepG2, HT1080, COS-7, WS-1	MTT assay, soft-agar assay, wound-healing assay, LDH release assay, AO/EtBr staining	- Potent activity against human cervical, liver, and fibrosarcoma cancer cells - Antineoplastic activity of 60% in HT1080 and HeLa cells - Antineoplastic activity of 50% in HepG2 cells	[130]
KF	1476	Marine mollusk (*Elysia rufescens*)	PC3, DU145, LNCaP, SKBR-3, BT474, MCF-7, MDA-MB-231, LoVo, MCF10A, HUVEC, HMEC-1, IMR90	Measurement of DNA synthesis, effect of BCL-2, MDR-1, and HER2/neu, cytotoxicity assay	- Potent activity against human prostate, breast, and colon cancer cells - A dose-dependent inhibition of DNA synthesis in DU145, PC3, LNCaP, MCF-7, SKBR-3, MDA-MB-231, BT474, LoVo at IC_50_ of 0.18, 0.07, 0.26, 0.28, 0.23, 0.39, 0.26, and 0.16 µM, respectively - A reduced inhibition of DNA synthesis in IMR90, MCF10A, HMEC-1, and HUVEC at IC_50_ of 3.13, 2.44, 1.88, and 1.62 µM, respectively	[131]

AO/EtBr: Acridine orange/ethidium bromide; A549: Type II pulmonary epithelial cells; BCL-2: B-cell lymphoma 2; BT474: Human breast cancer cells; BT549: Breast carcinoma cells; B16F10: murine melanoma cell line from a C57BL/6J mouse; Caco-2: Human colon cancer cells; COS-7: Cercopithecus aethiops kidney cells; DU145: Human prostate cancer cell lines; HCT15: Colon carcinoma cells; HeLa: Human cervix adenocarcinoma cells; HepG2: Human liver cancer cells; HER2/neu: Human epidermal growth factor receptor 2; HMEC-1: Human Mammary epithelial cells; HPLC: High-performance liquid chromatography; HT-29: Human colon cancer cells; HT1080: Human fibrosarcoma cells; HUVEC: Human umbilical vein endothelial cell line; IEC: Ion-exchange chromatography; IMR90: Normal lung fibroblast cells; IC50: The half maximal inhibitory concentration; KF: Kahalalide F; LC50: Lethal concentration that kills 50% of test population; LDH: Lactate dehydrogenase; LNCaP: Human prostate cancer cells; LoVo: Human colon (supraclavicular lymph node metastasis) cells; MCF-7: Human breast cancer cells; MCF10A: Human breast epithelial cell line; MDA-MB-231: Human breast cancer cells; MDR-1: Multidrug resistance mutation; MKN45: Human gastric carcinoma cells; MN-11: Mouse fibrosarcoma cells; MTT: 3-(4,5-dimethylthiazol-2-yl)-2,5-diphenyl tetrazolium bromide; MTS: 3-(4,5-dimethylthiazol-2-yl)-5-(3-carboxymethoxyphenyl)-2-(4-sulfophenyl)-2H-tetrazolium; NUGC-4: Signet-ring carcinoma cells; PC3: Prostate cancer cells; qRT-PCR: Real-Time Quantitative Reverse Transcription PCR; SKBR-3: Human breast cancer cell; UPLC-ESI-MS/MS: Ultra-performance liquid chromatography-electrospray ionization-mass spectrometry; U937: Human lymphoma cells; WS-1: Human kidney cells.

**Table 5:** Anticancer effects of bioactive peptides from marine animals—in vivo studies.

Peptide Name/Peptide Containing Compound	Size of Peptide (Da)	Source	Organism	Analysis	Major Findings	Reference
Pardaxin	-	Finless sole *(Pardachirus marmoratus)*	Syrian golden hamsters (5–6 weeks old)	Tumor volume measurement, PGE2 level assay	- Average tumor volume decreased from 131.4 mm^3^ to 36.2 mm^3^ - PGE2 level decreased from 800% to 500%	[132]
			20 C57BL/6 female mice (35 days old)	Analysis of GOT, GPT, BUN, Cre, UA, TCHO, TG, TBIL, TP, and ALB levels, cytokine expression levels, and immunohistochemistry	- Tumor volume decreased to 200 mm^3^ - No changes in body weight - No toxicity observed to normal cells - TNF-α expression of < 200 pg/mL - MIP-1α expression of < 2500 pg/mL - IL-1β expression of < 6000 pg/mL	[126]
Conus venom	-	Vexillum cone snail *(Conus vexillum)*	54 adult Swiss albino male mice (Average body weight: 20–25 g)	Lipid peroxidation inhibition assay, estimation of nitric oxide, CAT, Cu/Zn SOD, and LDH activity, TAC and GSH content	- GSH content decreased from 400 µg/g to 325 µg/g - CAT activity decreased from 0.35 U/mL to 0.27 U/mL - Cu/Zn SOD activity decreased from 30 U/mL to 20 U/mL - TAC content decreased from 0.2 mmol/L to 0.15 mmol/L - LDH activity increased from 295.53 ± 8.1 U/L to 875 ± 12.1 U/L in - More than 235% increase in MDA level as compared to control	[133]

ALB: Albumin; BUN: Blood urea nitrogen; CAT: Catalase; Cre: Creatinine; CU/Zn SOD: Copper/zinc superoxide dismutase; GOT: Glutamic oxaloacetic transaminase; GPT: Glutamic pyruvic transaminase; GSH: Glutathione; LDH: Lactate dehydrogenase; MDA: Malondialdehyde; PGE2: Prostaglandin E2; TAC: Total antioxidant capacity; TBIL: Total bilirubin; TCHO: Total cholesterol; TG: Triglyceride; TP: Total protein; UA: Uric acid.

**Table 6:** Anticancer effects of bioactive peptides from marine animals—clinical trials.

Peptide/Peptide Containing Compound	Source	Subject	Analysis	Major Findings	Phase	Reference
KF	Marine mollusk (*Elysia rufescens*)	38 cancer patients (23 male and 15 female; 27–73 years old)	Pharmacokinetic analysis	- A maximum tolerated dose of 800 µg/m^2^ - The recommended dose of 650 µg/m^2^ - Dose-limiting toxicities with weekly 1 h infusion; however, no accumulative cytotoxicoty observed - A 25–50% reduction in tumor size in metastatic lung adenocarcinoma patients	I	[134]
		32 cancer patients (49–81 years old)	Pharmacokinetic analysis	- The maximum tolerated dose of 930 µg/m^2^ per day - The recommended dose of 560 µg/m^2^ with a half-life of 0.47 h - Dose-limiting toxicities with 5 consecutive days treatment with 1 h infusion	I	[135]
		24 cancer patients (28–89 years old)	RECIST	- Average overall survival of 10.8 months - No toxicity to the normal cells observed	II	[136]
DOLA-10	Marine mollusk (*Dolabella Auricularia*)	22 cancer patients (10 male and 12 female; 27–72 years old)	Pharmacokinetic and pharmacodynamic analysis	- The maximum tolerated dose of 300 µg/m^2^ - A recommended dose of 400 µg/m^2^ for patients with minimal prior chemotherapy - A recommended dose of 325 µg/m^2^ for patients with > 2 chemotherapy	I	[137]
		10 NSCLC patients (female; 47–80 years old)	Physical examination, complete blood count, computed timigraphy, and liver function tests	- Only 3 patients had a stable disease - 2 patients with grade-4 neutropenia, 2 patients with grade-3 hyperglycemia, and 1 was hospitalized	II	[138]
		12 advanced melanoma patients (7 male and 5 female; 26–80 years old)	Pharmacokinetic analysis	- DOLA-10 in plasma showed total body clearance, and the distribution volume in the body volume at steady-state was 2.61 ± 1.9 L/h/m^2^ and 28.4 ± 13 L/m^2^	II	[139]

DOLA-10: Dolastatin-10; KF: Kahalalide F; NSCLC: Non-small-cell lung carcinoma; RECIST: Response evaluation criteria in solid tumor.
